# Repurposing the Antiemetic Metoclopramide as an Antiviral Against Dengue Virus Infection in Neuronal Cells

**DOI:** 10.3389/fcimb.2020.606743

**Published:** 2021-02-02

**Authors:** Ting-Jing Shen, Vu Thi Hanh, Thai Quoc Nguyen, Ming-Kai Jhan, Min-Ru Ho, Chiou-Feng Lin

**Affiliations:** ^1^Graduate Institute of Medical Sciences, College of Medicine, Taipei Medical University, Taipei, Taiwan; ^2^Department of Microbiology and Immunology, School of Medicine, College of Medicine, Taipei Medical University, Taipei, Taiwan; ^3^International Ph.D. Program in Medicine, College of Medicine, Taipei Medical University, Taipei, Taiwan; ^4^Centre for Hematology and Blood Transfusion, Bach Mai Hospital, Hanoi, Vietnam; ^5^Centre for Tropical Diseases, Bach Mai Hospital, Hanoi, Vietnam; ^6^Center of Infectious Diseases and Signaling Research, National Cheng Kung University, Tainan, Taiwan

**Keywords:** dengue virus, antiemetics, metoclopramide, antiviral, dopamine receptor

## Abstract

Dengue virus (DENV) is transmitted by Aedes mosquitoes to humans and is a threat worldwide. No effective new drugs have been used for anti-dengue treatment, and repurposing drugs is an alternative approach to treat this condition. Dopamine 2 receptor (D2R) is a host receptor positively associated with DENV infection. Metoclopramide (MCP), a D2R antagonist clinically used to control vomiting and nausea in patients with DENV infection, was putatively examined for inhibition of DENV infection by targeting D2R. In the mouse neural cell line Neuro-2a with D2R expression, a plaque assay demonstrated the antiviral efficacy of MCP treatment. However, in the cell line BHK-21, which did not express D2R, MCP treatment caused no further inhibition of DENV infection. Either MCP treatment or exogenous administration of a neutralizing D2R antibody blocked DENV binding. Treatment with MCP also reduced DENV dsRNA replication and DENV-induced neuronal cell cytotoxicity *in vitro*. An *in vivo* study demonstrated the antiviral effect of MCP against DENV-induced CNS neuropathy and mortality. These results showed that repurposing the D2R-targeting antiemetic MCP is a potential therapeutic strategy against DENV infection.

## Introduction

*Aedes* mosquitoes transmit four serotypes of dengue virus (DENV), namely, DENV1, DENV2, DENV3, and DENV4, to cause arthropod-borne viral diseases (Guzman et al., 2016; Ferguson, 2018) in tropical and subtropical countries. An estimated 390 million people are at risk of DENV infection annually (Guzman et al., 2010; Ferguson, 2018). Following receptor binding, the interaction with the viral envelope (E) protein allows the internalization of virus particles *via* clathrin-dependent and clathrin-independent endocytosis (Acosta et al., 2008; van der Schaar et al., 2008). Within the virus-containing endosomes, the pH value decreases to induce a shape change in the E protein of the virus, leading to fusion with the endosomal membrane. As a result, the viral single-strand positive-sense RNA enters the cytoplasm to induce viral protein translation as well as dsRNA replication (Zaitseva et al., 2010). Then, the virions are transported through the trans-Golgi network and secreted (Yu et al., 2008; Welsch et al., 2009) to generate the next infectious cycle. Targeting the DENV infectious process is speculated to be therapeutic against viral infection.

The symptoms of mild dengue fever include fever, rash, headache, and joint pain (Hadinegoro, 2012). However, other symptoms occurring in dengue fever, such as plasma leakage and abnormal hemostasis, may cause severe disease. In addition to dengue hemorrhagic fever (DHF) and dengue shock syndrome (DSS) (Leong et al., 2007), encephalitis and encephalopathy have been observed in severe dengue disease in clinical studies, which suggests that DENV indirectly affects the CNS to cause encephalopathy in DENV infection (Solomon et al., 2000). DENV can be identified in the cerebrospinal fluid and the brain tissue of encephalitis patients (Verma et al., 2014). It has been suggested that DENV can enter neuronal systems through membrane receptors and contribute to neurological manifestations, including encephalitis, encephalopathy, and dengue-associated neuromuscular complications (Calderon-Pelaez et al., 2019). Appropriate medical interventions are needed to prevent neurological complications and death.

Dopamine receptors, a class of G protein-coupled receptors that are highly expressed in the vertebrate central nervous system (CNS), mediate multiple functions in motivation, pleasure, cognition, memory, learning, and fine motor control (Ito et al., 1999). Additionally, dopamine 2 receptor (D2R) contributes to virus infection, including human immunodeficiency virus infection (Gaskill et al., 2014) as well as Japanese encephalitis virus infection (Simanjuntak et al., 2017), by promoting viral entry into cells at the early stage. Prochlorperazine (PCZ), a D2R antagonist, shows antiviral activity against DENV infection (Simanjuntak et al., 2014). Additionally, targeting D2R by another compound, chlorpromazine (CPZ), blocks DENV infection *in vitro* and *in vivo* (Ho et al., 2017). DENV causes infection in the brain and induces CNS neuropathy in severe dengue patients (Solomon et al., 2000; Verma et al., 2014), and treatment with repurposed dopamine blockers can inhibit DENV infection as an alternative antiviral strategy. Dopamine blockers include two groups, phenothiazines and benzamides. While PCZ and CPZ are phenothiazines, aminobenzamide is a benzamide derivative that can also inhibit viral proteases in the flavivirus DENV and West Nile virus (Aravapalli et al., 2012). Additionally, *N*-(adamantane-1-yl)-4-[(adamantane-1-yl)sulfamoyl]benzamide can inhibit DENV infection (Joubert et al., 2018). The antiemetic metoclopramide (MCP) is also a D2R-blocking benzamide derivative that is clinically used as a common drug for treating nausea and vomiting in dengue patients. In this study, we investigated the approach of repurposing metoclopramide as a D2R antagonist to inhibit DENV infection.

## Materials and Methods

### Chemicals, Cell Culture, Virus, and Reagents

MCP hydrochloride (4-amino-5-chloro-N-[2-(diethylamino)ethyl]-2-methylxo benzamide monohydrochloride; C_14_H_22_ClN_3_O_2_ • HCl) (Sigma-Aldrich, St. Louis, MO), a white crystalline, is dissolved in distilled water with a concentration of 100 mM and kept at −20°C. The optimal dose of MCP was evaluated by MTT and LDH assay. Murine neuroblastoma cell line Neuro-2a (ATCC; CCL-131) and baby hamster kidney cell line BHK-21 (ATCC; CCL-10) were maintained and cultured in medium DMEM with 10% fetal bovine serum (FBS) (Biological Industries, Israel), 100 U/ml penicillin, and 100 μg/ml streptomycin at 37°C and 5% CO_2_. *Aedes albopictus* C6/36 cells (ATCC; CRL-1660) were cultured in minimal essential medium (MEM) (Invitrogen, California, USA) with 10% FBS, 100 U/ml penicillin, 100 μg/ml streptomycin, 1% sodium pyruvate, 1% non-essential amino acids (Thermo Fisher Scientific) and 1% HEPES at 28°C and 5% CO_2_. The propagation of DENV serotype 2 (PL046) in C6/36 cells with multiplicity infection (MOI) of 0.01 was maintained at 28°C and 5% CO_2_ for 5 days. The supernatant was harvested and purified by centrifugation. Virus titers were evaluated by plaque assay on BHK-21 cells as described below. The antibodies used in the study are antibodies of DENV dsRNA, NS1, NS2B, NS3, NS4, and NS5 (GeneTex, San Antonio, TX) and Alexa Fluor 488-conjugated goat anti-mouse IgG (Invitrogen, Carlsbad, CA).

### Immunocytochemistry

For D2R detection, the cells were stained with primary antibody for 1 h at 4°C followed by incubation with the secondary antibody. The expression of D2R was analyzed using flow cytometry (Attune NxT, Thermo Fisher Scientific, Waltham, MA). For dsRNA detection, the cells were infected with DENV 2 (MOI = 1) for 6 h, the medium was removed, and the cells were washed and fixed with 4% paraformaldehyde. After 15 min, the cells were permeabilized and blocked with permeabilization buffer (PBS + 1% Triton X-100) at room temperature and blocking buffer (PBS + 1% BSA + 0.01% Triton X-100) at 4°C, respectively. Then, cells were stained with mouse anti-dsRNA J2 primary antibody overnight at 4°C followed by incubation with the Alexa Flour 488-conjugated goat anti-mouse secondary antibody (Thermo Fisher Scientific) and DAPI (Sigma-Aldrich) at room temperature for 15 min. The expression of dsRNA was visualized under a fluorescence microscope (EVOS FL cell imaging system, Thermo Fisher Scientific).

### DENV Infection and Treatment

The Neuron-2a and BHK-21 cells were infected with DENV 2 (MOI = 1) at 37°C with or without MCP. After 2 h, the virus was removed from wells and cells supplied by the medium with or without MCP. Then, the cells were incubated at 37°C in 5% CO_2._ At indicated time points, sampled were collected to conduct further analysis.

### Plaque Assay

BHK-21 cells were seeded in 12-well plates at a density of 7×10^4^ cells per well and then inoculated with the supernatants for treatment at diluted ratios of 10^-1^, 10^-2^, and 10^-3^. After 2 h, the virus inoculum was discarded and replaced with a new DMEM containing 4% FBS and 0.5% methylcellulose. Five days after infection, the methylcellulose was discarded and washed with PBS twice. The cells were fixed and stained with crystal violet solution containing 1% crystal violet, 0.64% NaCl, and 2% formalin. The next day, the stain was removed by water, and the plates were kept dry at room temperature; the viral plaques were counted by eye observation.

### Western Blotting

Treated Neuro-2a and BHK-21 cells were collected and lysed. The protein samples were separated by electrophoresis in 10% SDS-PAGE and transferred to nitrocellulose membrane and analyzed to determine the presence of the NS1, NS3, NS4B, and NS5 proteins according to our previous works (Ho et al., 2017). The use of ImageJ image software quantified the band density of western blot.

### Fluorescent DENV

The purified DENV was labeled with fluorescent (Alexa Fluor 549) accordingly (Ho et al., 2017). The infected cells were visualized under a fluorescence microscope (EVOS FL cell imaging system).

### Cell Viability and Cytotoxicity Assay

Cell viability and cytotoxicity were determined *in vitro* by using a colorimetric 3-(4,5-Dimethylthiazol-2-yl)-2,5-Diphenyltetrazolium Bromide (MTT) assay (Bio Basic, Toronto, Canada) and a Cytotoxicity Detection kit assay (Roche Diagnostics, Lewes, UK) (Ho et al., 2017), respectively.

### Animal Model

As approved by the Institutional Animal Care and User Committee of the National Defense Medical Center (IACUC number: 16-261) and based on the protocol of DENV infection *in vivo* (Tsai et al., 2016; Ho et al., 2017; Shen et al., 2019), seven-day-old Institute of Cancer Research (ICR) strain suckling mice were inoculated intracerebrally with 2.5×10^5^ plaque-forming units (PFU) and intraperitoneally with 7.5×10^5^ PFU of DENV 2 (PL046). MCP (1 mg/kg) was given concurrently by intracerebral and intraperitoneal injections. The time-kinetic changes in disease progression and survival rate of mice were monitored and recorded daily. The disease severity was scored as follows: 0 for healthy; 1 for minor illness, including weight loss, reduced mobility, and a hunchback body orientation; 2 for limbic seizures; 3 for moving with difficulty and anterior limb or posterior limb weakness; 4 for paralysis; and 5 for death. Organs were harvest homogenized in PBS at day 9 post-infection. The protein expressions and tissue viral load were examined by Western blotting and plaque assay, respectively.

### Statistical Analysis

The data were analyzed as the mean ± standard deviation (SD) and compared by the Unpaired *t*-test as well as one-way ANOVA (Tukey’s multiple comparisons test) by using GraphPad Prism software. The survival rate followed a log-rank test. Statistical significance was set at *p <* 0.05.

## Results

### Expression of D2R on the Surface of Neuro-2a and BHK-21 Cells and Its Roles in Mediating DENV Infection

Targeting dopamine receptor D2 (D2R) hinders DENV infection in neuronal cells *in vitro* and in the brain *in vivo* (Simanjuntak et al., 2014; Ho et al., 2017). For the identification of FDA-approved D2R antagonists for anti-dengue treatment, the antiemetic MCP was individually investigated in this study because it has been used not only for D2R antagonism but also for symptomatic medication, including persisting nausea and vomiting, during the onset of dengue fever (Marra et al., 2011). In this study, repurposing anti-nausea/vomiting MCP as an anti-dengue strategy was examined.

**Figure 1:**
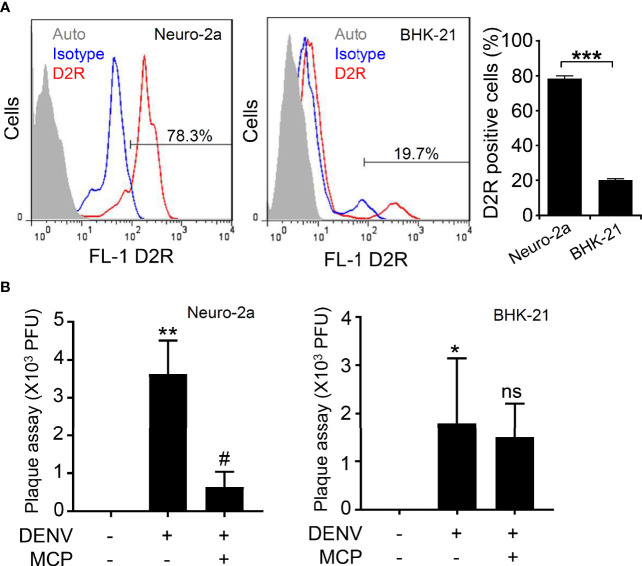
Expression of dopamine receptor D2 (D2R) and its roles in mediating dengue virus (DENV) infection in the murine neural cell line Neuro-2a. **(A)** Representative histograms and statistical results of D2R expression, as measured by flow cytometry, show the percentages of D2R expression in the Neuro-2a and BHK-21 cells used in this study. Auto, autofluorescence. Isotype, isotype control of IgG. **(B)** In the presence of the D2R antagonist MCP, plaque assays showed the level of viral replication 24 h post-infection in the Neuro-2a and BHK-21 cells. The quantitative data are depicted as the mean ± SD of three independent experiments. *p < 0.05, **p < 0.01, and ***p < 0.001, compared with the untreated cells. ^#^*p <* 0.05, compared with DENV. ns, not significant compared with DENV.

D2R-expressing cells are needed to prove the antiviral efficacy of MCP. Based on our previous study showing that DENV infects Neuro-2a and BHK-21 cells (Ho et al., 2017), the expression of D2R was monitored. Nonfixed immunostaining followed by flow cytometric analysis showed that Neuron-2a cells highly expressed D2R at 78.3% **(****Figure 1A**, left), while BHK-21 cells displayed a relatively decreased level of D2R expression (19.7%) **(****Figure 1A**, right). Based on these results, Neuro-2a cells were subsequently used in this study.

For antiviral treatment, an optimal dose of MCP without lethal effects is necessary. Using MTT and LDH assays, we evaluated serial concentrations of MCP from 1 to 2,000 µM to monitor its potential effects on cell growth and cytotoxicity. The MTT assay showed a significant inhibitory effect of MCP on cell growth at concentrations over 200 µM **(****Supplemental Figure S1A****)**. At concentrations between 800 and 2,000 µM, the LDH assay displayed significant cytotoxic effects of MCP **(****Supplemental Figure S1B****)**. According to the results, 200 µM of MCP was susceptible to use for further examining its antiviral efficacy in Neuro-2a cells.

To measure the antiviral efficacy of MCP, we performed a plaque assay, which showed that the viral titers after MCP treatment of Neuro-2a cells were significantly decreased **(****Figure 1B**, left). However, MCP did not cause effects on BHK-21 cells, probably due to the lower expression of D2R in BHK-21 cells **(****Figure 1B**, right). Based on these data, MCP can impede DENV infection in D2R-positive Neuro-2a cells.

### MCP Treatment as an Antiviral Strategy Against Viral Protein Expression and dsRNA Replication

**Figure 2:**
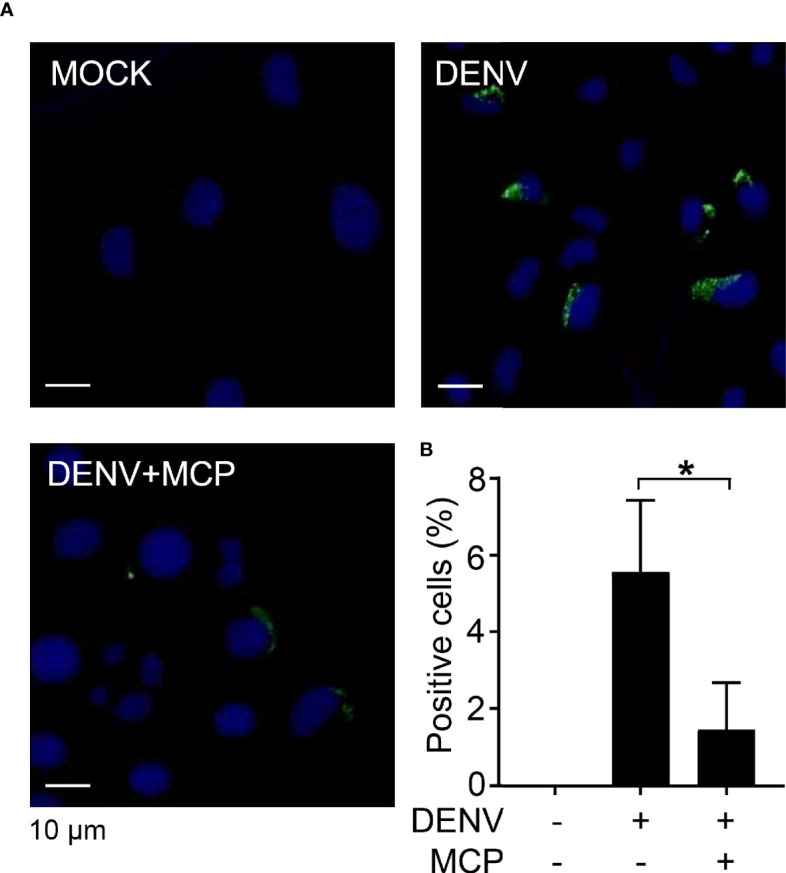
Metoclopramide (MCP) treatment abolishes dengue virus 2 (DENV2) dsRNA replication. Neuro-2a cells were inoculated with DENV2 (MOI = 1) for 6 h in the presence of MCP. **(A)** Immunocytochemistry and **(B)** the percentages of viral dsRNA (*green*) were shown. DAPI (*blue*) was used for nuclear staining. The quantitative data are depicted as the mean ± SD of three independent experiments. *p < 0.05.

To investigate the possible antiviral actions of MCP, we explored the cellular responses and infectious processes during DENV infection. The Western blot results showing the inhibition of DENV NS1, NS3, NS4B, and NS5 protein expression in the presence of MCP confirmed the antiviral effect of MCP in the DENV-infected Neuro-2a cells **(****Supplemental Figure S2A****)** but not in the DENV-infected BHK-21 cells **(****Supplemental Figure S2B****)**. Immunocytochemistry followed by fluorescent microscopic observation **(****Figure 2A****)** showed the expression of dsRNA in the DENV-infected Neuro-2a cells, while MCP treatment significantly reduced its expression **(****Figure 2B****)**. These results confirm the antiviral efficacy of MCP against DENV protein synthesis as well as dsRNA replication.

### MCP Treatment as an Antiviral Strategy Against D2R-Mediated Viral Binding/Entry Does Not Affect the Antiviral IFN Response

**Figure 3:**
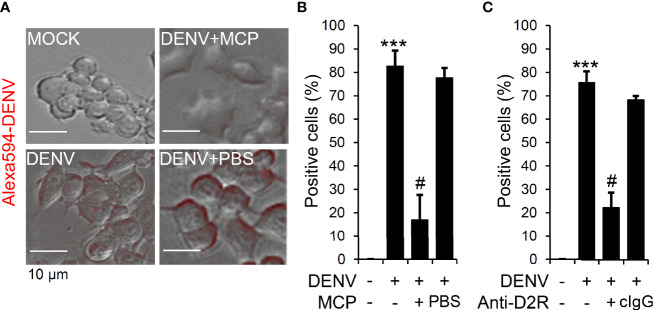
Metoclopramide (MCP) or neutralizing anti-D2R treatment hinders dengue virus (DENV) entry. **(A)** Fluorescence microscopy measured the positive Neuro-2a cells carrying Alexa-594-labeled (*red*) DENV 2 (MOI = 1) 2 h post-infection in the presence of MCP or the neutralizing anti-D2R IgG. **(B** & **C)** represented the counts of Alexa-594-labeled (*red*) DENV 2-positive cells. PBS and isotype control IgG (cIgG) were used as controls. The quantitative data are depicted as the mean ± SD of three independent experiments. ***p < 0.001, compared with the untreated cells. ^#^*p <* 0.05, compared with DENV.

Viral binding/entry is an early step for DENV infection (Cruz-Oliveira et al., 2015). D2R is highly expressed in Neuro-2a cells; therefore, we next evaluated the MCP-mediated blockade of DENV binding/entry. Fluorescent DENV was prepared according to our previous works (Ho et al., 2017). Microscopic observation **(****Figure 3A****)**, as well as quantification analysis **(****Figure 3B****)**, showed viral binding/entry at 2 h post-inoculation, which was significantly impeded by MCP treatment. To further validate the requirement of D2R in facilitating DENV binding/entry in Neuro-2a cells, we exogenously administered neutralizing antibodies against D2R. The results demonstrated a significant inhibitory effect of D2R neutralization on DENV entry/binding **(****Figure 3C****)**. Our findings demonstrate the MCP-mediated blocking of D2R-mediated viral entry/binding at the early stage of DENV infection.

To confirm the target of MCP underlying its antiviral capacity through interference with viral binding/entry, we monitored the off-target effect of MCP on the type I IFN response to assess its potent antiviral activity. The results of ELISA showed a significant increase in IFN-β production in the DENV-infected Neuro-2a cells; however, MCP did not increase IFN-β production **(****Supplemental Figure S3****)**. These data indicate that MCP directly blocked D2R-mediated viral binding/entry independent of the antiviral type I IFN response.

### MCP Treatment Reduces the *In Vitro* Neurotoxicity Induced by DENV Infection

**Figure 4:**
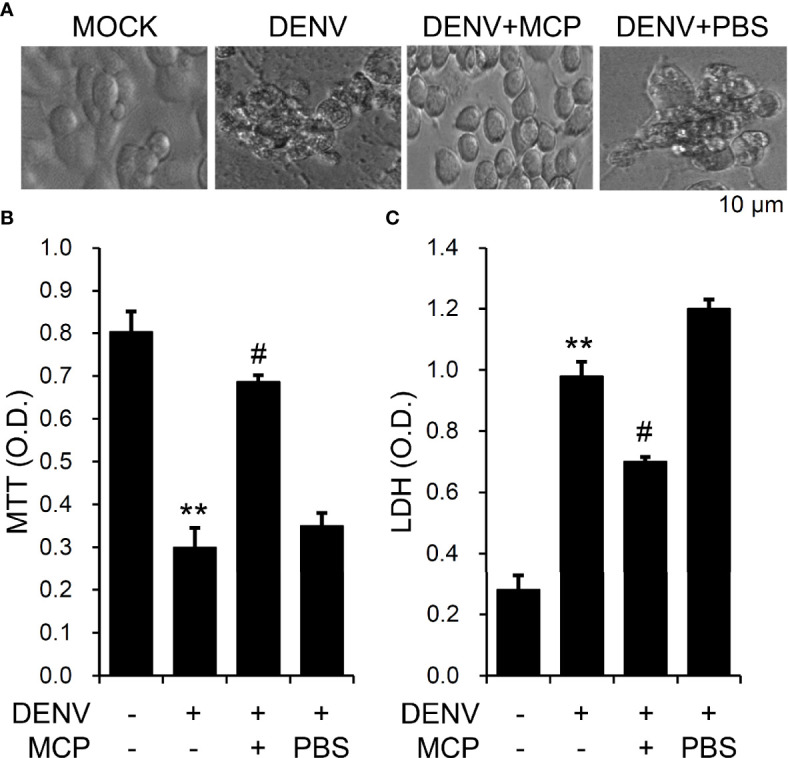
Metoclopramide (MCP) treatment reduces dengue virus (DENV)-induced neurotoxicity *in vitro*. Following pretreatment with MCP, **(A)** cell morphology, **(B)** MTT, and **(C)** lactate dehydrogenase (LDH) cytotoxicity detection assays showed cell growth, viability, and cytotoxicity, respectively, in the DENV-infected cells at 72 h. PBS was used as the control. For all images, representative data were selectively obtained from three individual experiments. All quantitative data are shown as the mean ± SD of three independent experiments. **p < 0.01, compared with the untreated cells. ^#^*p <* 0.05, compared with DENV.

Infection with DENV causes neuronal cell apoptosis, particular 72 h post-infection (Ho et al., 2017). To analyze the roles of the D2R pathways involved in DENV infection in neuronal cells, we showed that pretreatment with MCP effectively abolished the DENV-induced changes in cell morphology **(****Figure 4A****)**, cell growth inhibition **(****Figure 4B****)**, and cytotoxicity **(****Figure 4C****)**. These results indicate the cytoprotective effects of MCP, which targets D2R-mediated DENV binding/entry on DENV-induced neurotoxicity *in vitro*.

### MCP Treatment Abolishes DENV Infection and Acute Viral Encephalitis-Like Disease Progression *In Vivo*

**Figure 5:**
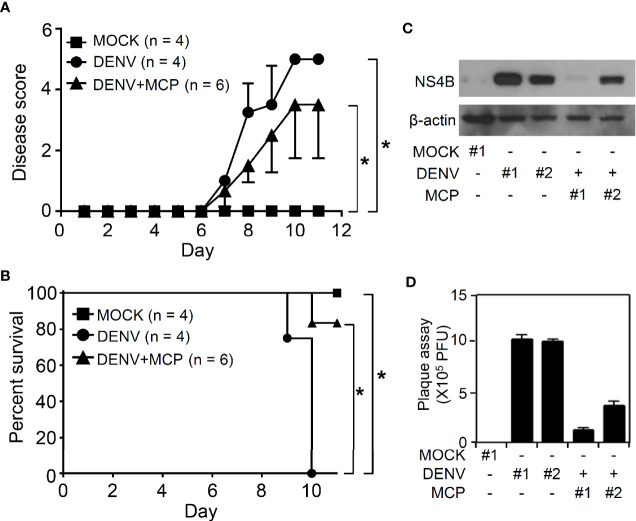
Metoclopramide (MCP) treatment delays the clinical scores in suckling mice during dengue virus (DENV) infection. Seven-day-old ICR suckling mice were inoculated with DENV 2 by concurrent intracranial and intraperitoneal injections with or without MCP (1 mg/kg) cotreatment. Time-kinetic changes in **(A)** the clinical scores and **(B)** the survival rates of mice were measured (MOCK = 4, DENV = 4, DENV+MCP = 6). **(C)** Western blot analysis of viral NS4B protein expression and **(D)** plaque assays of viral replication in the brains of the ICR suckling mice at 9 days post-infection were shown (MOCK = 1, DENV = 2, DENV+MCP = 2). Each bar represented a mouse sample (triplicate analyses). *p < 0.05.

We next verified the *in vivo* antiviral effects of MCP against viral replication, viral encephalitis, and mortality in DENV-infected ICR suckling mice (Ho et al., 2017; Shen et al., 2019). For this animal study, we inoculated seven-day-old ICR suckling mice by intracerebrally and intraperitoneally given with 2.5 × 10^5^ PFU and 7.5 × 10^5^ PFU of DENV 2, respectively. Mice were concurrently intracranially and intraperitoneally treated with MCP (dissolved in PBS) in a dose of 1 mg/kg body weight. A significant increase in the clinical scores **(****Figure 5A****)** occurred in the DENV-infected mice compared to the mock-infected mice by day 7 post-infection. The survival rate of DENV-infected mice decreased by day 9 post-infection, and all of the mice died by day 10 post-infection **(****Figure 5B****)**. Cotreatment with MCP significantly reduced DENV-induced disease progression and mortality. According to the Western blot analysis of the NS4B viral protein **(****Figure 5C****)** and the plaque assays for detecting the production of infectious particles **(****Figure 5D****)**, DENV caused significant infection and replication in the mouse brains at day 9 post-infection, and MCP inhibited viral protein expression and replication. These data indicate that a single-dose treatment of MCP partly abolished encephalitic DENV infection in our model, which leads to neural impairment following viral replication.

## Discussion

The number of cases of DENV infection is continuously increasing in several areas of the world (Guzman et al., 2010; Tuiskunen Bäck and Lundkvist, 2013). The identification of antiviral drugs is still urgently needed. To date, there have been no effective drugs for the treatment of DENV infection. Drug repurposing has been widely studied to find new effects of existing drugs that contribute to inhibiting DENV infection (Botta et al., 2018). This study showed the antiviral activity observed through repurposing an antiemetic medication.

Pharmacologically targeting D2R by using selective antagonists, including PCZ and CPZ, decreases DENV infection and viral replication in neuronal cells *in vitro* and in the brain *in vivo* (Simanjuntak et al., 2014; Ho et al., 2017). Notably, during the onset of dengue fever as well as dengue hemorrhagic fever, the antiemetic MCP is used to alleviate the symptoms, including persisting nausea and vomiting (Marra et al., 2011). However, its potential antiviral efficacy through pharmacological targeting of D2R has not been addressed. In this study, repurposing the FDA-approved antiemetic MCP as an alternative D2R antagonist was evaluated for its potential application as an anti-dengue strategy. We provided evidence to determine the antiviral effect of MCP against DENV infection *in vitro* and *in vivo*. By using our previous experimental murine model of CNS impairment accompanied by CNS inflammation (Tsai et al., 2016; Ho et al., 2017; Shen et al., 2019), MCP treatment significantly reduced DENV-induced progressive neuropathic symptoms, including reduced mobility, a hunchback body orientation, limbic seizures, paralysis; and mortality. For MCP’s therapeutic advantages, this work also confirmed the possible target and protective roles of MCP in blocking DENV infection as well as DENV-induced neurotoxicity. The potential neuroprotection either by improving neuronal cell survival or by inducing anti-inflammation in CNS needs validation by using an advanced therapeutic protocol.

Similar to PCZ and CPZ, MCP is also a dopamine antagonist and is commonly used to treat nausea and vomiting (Welliver, 2014; Athavale et al., 2020). However, PCZ and CPZ were shown to be effective in treating DENV infection by targeting clathrin-mediated endocytosis as well as D2R (Simanjuntak et al., 2014; Ho et al., 2017). In contrast to PCZ and CPZ, MCP belongs to the benzamide group. Some benzamide derivatives have an inhibitory effect on DENV infection (Welliver, 2014), and an aminobenzamide scaffold can be utilized to inhibit DENV and West Nile virus protease (Aravapalli et al., 2012). There are currently no further reports showing the ability of MCP to inhibit DENV. Based on these rationales, we hypothesize that MCP can retard DENV infection, similar to drugs used for D2R blocking.

According to the D2R expression, we selected the Neuro-2a cell line with a high expression of D2R as a target cell for studying the effect of MCP treatment on DENV infection. The envelope proteins on the virus and proteins on the surface of the cell interact during virus infection. Thus, blocking virus attachment and trafficking into cells has become an attractive strategy to prevent DENV infection (De La Guardia and Lleonart, 2014). Previously, the inhibitory effect of PCZ was shown to avoid DENV infection by targeting D2R and the clathrin-coated vesicle pathway (Simanjuntak et al., 2014). Similarly, CPZ can block DENV infection by targeting D2R (Ho et al., 2017). By using DENV labeled with Alexa 594 fluorescence to assess the ability of MCP to inhibit DENV attachment and entry into cells, we showed that either MCP or a neutralizing antibody against D2R could inhibit DENV binding and entry into Neuro-2a cells. These results indicated the blockade of DENV infection by using the D2R-targeting MCP. HIV replication in macrophages is enhanced through activation of D2R (Gaskill et al., 2009; Gaskill et al., 2014), and signaling of D2R facilitates JEV infection (Simanjuntak et al., 2017). Repurposing MCP can be used as an antiviral strategy against not only DENV but also HIV and JEV infection.

Previous works identified the induction of neurotoxicity by DENV *in vitro* (Despres et al., 1998; Jan et al., 2000; Ho et al., 2017) and *in vivo* (de Miranda et al., 2012; Velandia-Romero et al., 2012). However, the cytotoxic mechanisms are putatively varied during the infectious processes of viral attachment, internalization, endocytosis, viral protein synthesis, virion assembly, and release. Severe dengue patients may develop symptoms associated with neurological complications, threatening the patient’s life (Despres et al., 1998; Solomon et al., 2000). While the expression of dopamine receptors shows a characteristic pattern in neuronal cells, targeting dopamine receptors such as D2R (Simanjuntak et al., 2014; Ho et al., 2017) and D4R (Smith et al., 2014) is believed to have an anti-dengue effect, preventing infection as well as protecting against neurotoxicity in the CNS.

As a potential antiviral approach by repurposing MCP treatment, its immunoregulatory role remains unclear. The IFN-triggered antiviral responses are crucial for eliminating viral replication and spread during DENV infection (Liang et al., 2011; Sprokholt et al., 2017). Also, type 1 IFNs could block DENV viral translation in a protein kinase R-independent pathway to inhibit the infection (Diamond and Harris, 2001). As demonstrated in the experimental animal models, the immunocompromised AG129 mice lacking interferon-α/β and -γ receptors are more susceptible to DENV infection (Shresta et al., 2004; Milligan et al., 2015; Milligan et al., 2017). Our study represented that MCP treatment could reduce DENV infection but does not affect antiviral IFN type I response in Neuro-2a cells *in vitro*, suggesting its main antiviral effect on the blockade of the D2R-mediated viral binding/entry to the host cells. Accordingly, the limitation of the use of MCP-based antiviral therapy is D2R-dependent.

In conclusion, we evaluated the effectiveness of MCP, which is used to prevent nausea and vomiting symptoms in patients with dengue infection, on the blockade of DENV infection through a mechanism involving a D2R-mediated pathway. The results showed that MCP could inhibit DENV infection by interfering with virus binding/entry in a D2R-targeting manner. The results suggest that further research on the effect of this drug against DENV infection should be performed.

## Data Availability Statement

The original contributions presented in the study are included in the article/**Supplementary Material**. Further inquiries can be directed to the corresponding author.

## Ethics Statement

The animal study was reviewed and approved by the Institutional Animal Care and User Committee of the National Defense Medical Center (IACUC-18-088).

## Author Contributions

T-JS, VH, and TN performed most of the experiments and interpreted the results. C-FL participated in the design and supervision of the projects. M-RH and M-KJ conducted mouse experiments. T-JS and C-FL designed the concept of the project and wrote the manuscript. All authors contributed to the article and approved the submitted version.

## Funding

This work was supported by the grants from the Ministry of Science and Technology (MOST107-2321-B-038-001, 108-2320-B-038-026, and MOST109-2320-B-038-050) and the intramural funding 106TMU-CIT-01-2, Taipei, Taiwan.

## Conflict of Interest

The authors declare that the research was conducted in the absence of any commercial or financial relationships that could be construed as a potential conflict of interest.
