# Supplementary material for: Repurposing the Antiemetic Metoclopramide as an Antiviral Against Dengue Virus Infection in Neuronal Cells
Source: Front Cell Infect Microbiol. 2021 Feb 2;10:606743. doi: 10.3389/fcimb.2020.606743 (PMC7902071; doi:10.3389/fcimb.2020.606743)

Repurposing the antiemetic metoclopramide as an antiviral against dengue virus infection in neuronal cells

Supporting Information

Supporting Figure Legends

Figure S1. Effects of MCP on cell growth and cytotoxicity. (**A**) MTT assays showed the dose-dependent effects of MCP on the cell growth of Neuro-2a cells. (**B**) Lactate dehydrogenase (LDH) assays showed the cytotoxicity in MCP-treated Neuro-2a cells at different concentrations. OD, optical density. The quantitative data are depicted as the mean ± SD of three independent experiments. * $p < 0.05$ and ** $p < 0.01$. ns, not significant.

Figure S2. MCP treatment inhibits DENV2 infection. The Western blotting analysis showed viral protein expression during viral infection at 24 hours post-infection in (**A**) the Neuro-2a and (**B**) BHK-21 cells inoculated with DENV2 (MOI=1) in the presence of MCP. The clathrin inhibitor chlorpromazine (CPZ; 5 ng/ml) was used as a positive control. β-actin served as an internal control.

Figure S3. MCP treatment does not enhance IFN-β production. ELISA analysis showing IFN-β production in the DENV2 (MOI = 1)-infected Neuro-2a cells for 24 hours with or without MCP pretreatment. PBS was used as the solvent control. OD, optical density. The quantitative data are depicted as the mean ± SD of three independent experiments. ** $p < 0.01$, compared with the untreated cells. ns, not significant compared with DENV.

Shen et al Supplemental Figure 1

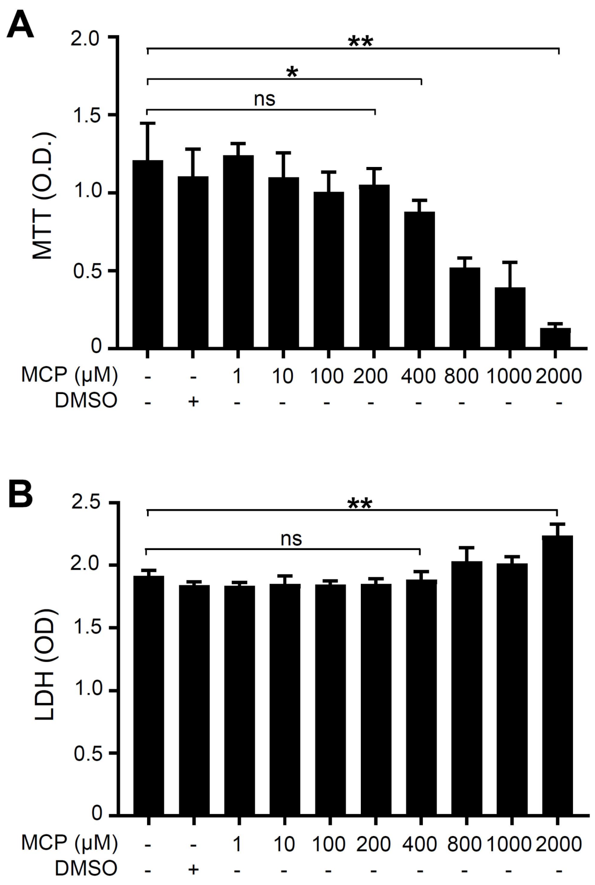

Shen et al Supplemental Figure 2

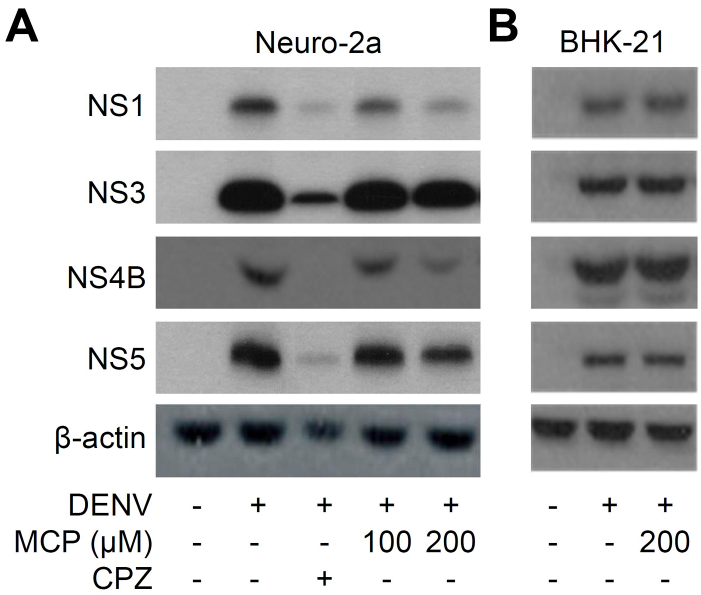

Shen et al Supplemental Figure 3

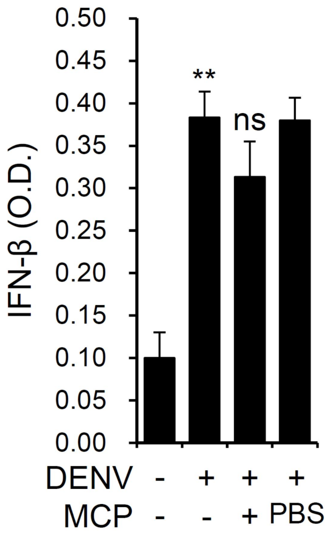

Supplement: Supplementary file 1 [file DataSheet_1.pdf]
